# Human platelets and their capacity of binding viruses: meaning and challenges?

**DOI:** 10.1186/s12865-015-0092-1

**Published:** 2015-04-28

**Authors:** Adrien Chabert, Hind Hamzeh-Cognasse, Bruno Pozzetto, Fabrice Cognasse, Mirta Schattner, Ricardo M Gomez, Olivier Garraud

**Affiliations:** EA3064—GIMAP, Université de Lyon, 42023 Saint-Etienne, France; Service des Agents infectieux et d’Hygiène, CHU de Saint-Etienne, 42055 Saint-Etienne, France; EFS Auvergne-Loire, 42023 Saint-Etienne, France; Laboratorio de Trombosis Experimental, Instituto de Medicina Experimental, ANM-CONICET, Buenos Aires, Argentina; Laboratorio de Virus Animales, Instituto de Biotecnología y Biología Molecular, UNLP-CONICET, La Plata, Argentina; Institut National de la Transfusion Sanguine, 75015 Paris, France; INTS, 6 rue Alexandre-Cabanel, 75015 Paris, France

**Keywords:** Platelets, Receptors, Viruses, Infection, Hemostasis

## Abstract

Blood platelets are first aimed at ensuring primary hemostasis. Beyond this role, they have been acknowledged as having functions in the maintenance of the vascular arborescence and, more recently, as being also innate immune cells, devoted notably to the detection of danger signals, of which infectious ones. Platelets express pathogen recognition receptors that can sense bacterial and viral moieties. Besides, several molecules that bind epithelial or sub-endothelial molecules and, so forth, are involved in hemostasis, happen to be able to ligate viral determinants, making platelets capable of either binding viruses or even to be infected by some of them. Further, as platelets express both Fc-receptors for Ig and complement receptors, they also bind occasionally virus-Ig or virus-Ig-complement immune complexes. Interplays of viruses with platelets are very complex and viral infections often interfere with platelet number and functions. Through a few instances of viral infections, the present review aims at presenting some of the most important interactions from pathophysiological and clinical points of view, which are observed between human viruses and platelets.

## Introduction

There is a large body of evidence that several types of viral infections led to sometimes severe and even life-threatening bleeding. In some of those infections, the platelet count drops dramatically; however, the precise mechanisms involved either in platelet destruction or impairment are still largely unclear, as well as the relative pathology counterpart of platelets and vascular endothelial cells. More largely, thrombocytopenia is common during or after viral infections and several mechanisms have been proposed to contribute to the drop of platelet count, including platelet destruction mediated by platelet-associated immunoglobulin G (IgG) or platelet–leukocyte aggregation, possibly leading to sequestration by macrophages, sequestration of platelets in the enlarged spleen, impaired production of thrombopoiesis, and direct effect of viruses on platelets.

**Figure 1:**
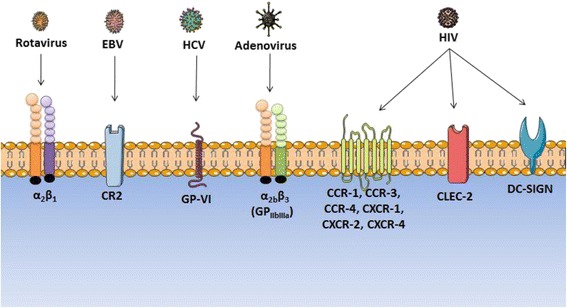
Platelet receptors acknowledged to bind viruses, along with their primary molecular targets. CLEC-2: C-type lectin-like type II transmembrane receptor. CR-2: Complement Receptor type 2. CCR-1, CCR-3, CCR-4: C-C chemokine Receptor type 1, 3 and 4. CXCR-1, CXCR-2, CXCR-4: C-X-C chemokine receptor type 1, 2 and 4. DC-SIGN: Dendritic Cell-Specific Intercellular adhesion molecule-3-Grabbing Non-integrin. GP-VI: Glycoprotein VI. PAR-1/PAR-4: Platelet Activating Receptor ¼.

Besides, albeit apparently infrequent though poorly examined in depth, platelets may bind and internalize infectious agents, with little clue on the outcome of the agent and of the “infected” platelet. The presence of viruses in platelets and their capacity of entry within those cells has been acknowledged for more than half a centenary: as early as of 1959, influenza viruses were forced to enter platelets experimentally, offering beautiful electronic microscopic images [1]. Questions relative to this situation have not been addressed then, perhaps because platelets were not yet acknowledged as “cells” but as debris. Viruses indeed find panoply of receptors that are not specific but rather permissive (as cartooned in Figure 1). FcγR bound viruses can also enter platelets as those cells can ingest immune complexes [2]. However, it is not yet clear whether platelets, by taking up viruses, contribute to the transport and the dissemination of infection *in vivo* or if, on the contrary, they help the host organism to defend against infection. Platelet–virus interactions may have an ambivalent role, depending on the virus species and on the platelet and megakaryocyte (MK) environment [3].

This mini-review aims at addressing the apparent complex issue of mutual relationships between certain viruses and platelets.

### Platelets and human immunodeficiency virus type 1 (HIV-1)

HIV-1 is a single positive-stranded RNA virus belonging to the *Retroviridae* family*,* genus *Lentivirus.* It was first visualized in 1990 in platelets and MKs of experimentally-infected mice [4]. In 2002, Youssefian et al. consistently identified HIV-1 in human MKs and platelets [5]. Several studies have thus clearly indicated that, during infection with HIV-1, there is a direct interaction between the virus, MKs and platelets; indeed, intact HIV-1 particles enclosed in endocytic vesicles were found to be sheltered from platelet secretory products while, when located in the surface connected canalicular system (SCCS), they were in contact with platelet secreted proteins [6]. These observations indicate that (1) platelets are able to endocytose HIV-1 virions; (2) HIV-1 can be enclosed within platelets, sheltered from host immune system aggression and transported by circulating platelets within the entire human body; and (3) viruses can come into contact with platelet-secreted alpha-granule contents leading to their destruction. In platelets incubated with HIV-1, characteristic endocytic vacuoles were identified close to the plasma membrane, tightly surrounding 1 or 2 viral particles. Examination of platelets from a patient with acquired immunodeficiency syndrome and high viremia suggested that HIV-1 endocytosis may also occur *in vivo* [5].

Regarding the receptors that favor HIV-1 entry in MKs and platelets, it has been found that MKs express the CD4 receptor for HIV-1 [7] as well as the co-receptors CXCR1, 2, 4, and CCR3 [8]. CXCR4+ MKs have been shown to be able to transfer CXCR4 to CXCR4 negative cells, rendering them capable of being infected by HIV-1 [9]. Platelets do not, however, express CD4 but only bear the HIV-1 co-receptors CXCR1, 2, 4 and CCR 1,3 and 4 [10-13]. Dendritic cell-specific ICAM-grabbing non-integrin (DC-SIGN), a C-type lectin receptor present on both macrophages and dendritic cells, has also been identified on platelets [6], with the capacity of binding HIV-1 on platelets [6,14]. Platelets also express the C-type lectin-like receptor 2 (CLEC-2). Further, a combination of DC-SIGN and CLEC-2 inhibitors strongly reduced the association of HIV-1 with platelets, indicating that these lectins are required for efficient HIV-1 binding to platelets [14].

Besides its entry in platelets, HIV-1 can activate infected as well as non-infected—neighboring—platelets. Indeed, HIV-1 trans-activator of transcription protein (Tat) directly interacts with platelets and induces platelet activation resulting in platelet micro-particle (PMP) release. This activation by Tat requires the chemokine receptor CCR3 and β3-integrin expression on platelets, as well as calcium flux. Tat-activated platelets secrete CD154 (CD40L) [15,16]. In general, enhanced platelet activation in HIV-1-infected patients seems a hallmark of the infection progression, as it strongly correlates with plasma viral load. Indeed, HIV-1 and platelet interactions engage the natural platelet partner that is vascular endothelium: levels of soluble endothelial activation markers (sVCAM-1, sICAM-1, and von Willebrand factor) are elevated in HIV-1 infected patients, and this elevation may be associated with a thrombotic state [7]. As stated, HIV-1 infection potentiates the pro-inflammatory potential of platelets: CD40L and HIV-1 Tat have been reported to synergize and contribute to inflammation [17]. Soluble CD40L also promotes the formation of complexes between inflammatory monocytes and activated platelets, which are detected by flow cytometry as monocytes::platelet doublets, which are positive for both CD14 and CD61 [18]. On the contrary, platelets can also secrete CXCL4/PF4 that is supposed to inhibit HIV-1 infection of T cells [19,20]; this exemplifies the complex relationship and interactions of platelets and viruses (for instance HIV1). This (PF4) platelet-associated chemokine indeed blocks HIV-1 entry and neutralizes HIV-1 activity, that suggests a role for platelets in the defense against HIV-1 infection. However, platelet-derived PF4 facilitates HIV-1 replication in human macrophage [21], and potentiates HIV activity [22]; this demonstrates that platelets products may exert dual actions regarding HIV infection, an example of definitely complex platelet::virus interaction.

Further, the clearance of senescent platelet by macrophages and neutrophils operates via P-Selectin/PSGL-1 [23]; platelets with bound-HIV seem to use the same pattern, which indicates that HIV uses platelets for protect itself from the immune system; however, since the first descriptions of HIV interactions with platelets, there has been no substantiated indication of productive replication of virus within platelets and by extend of a so-called platelet infection with HIV [5]; there is neither any evidence that HIV induces platelets to enhanced apoptosis.

A considerable reduction in the incidence of severe thrombocytopenia after the introduction of highly active antiretroviral therapy (HAART) was found, probably due to its ability to limit bone marrow damage induced by uncontrolled HIV-1 replication and opportunistic infections [24]. However, protease inhibitors or non-nucleoside reverse-transcriptase inhibitors do not reduce levels of the soluble platelet activation markers P-selectin and CD40L [25]. HAART alters inflammation linked to platelet cytokines in HIV-1-infected patients [26].

In all, HIV-1 is capable of entering platelets but outcome of this entry is still largely unclear, as platelets are not thought to consistently disseminate infection, nor are their considered to be reservoirs. HIV-1 also activates infected as well as non-infected platelets and contributes to local vascular inflammation. However, antiretroviral therapy—if it ameliorates the platelet decrease in numbers—accelerates the pro-inflammatory potential of platelets on vascular endothelium.

### Platelets and Hepatitis C virus (HCV)

HCV is a positive-sense single-stranded RNA virus of the *Flaviviridae* family, genus Hepacivirus*.* Chronic HCV infection is associated with the development of several extra-hepatic manifestations including thrombocytopenia [27]. When HCV treatment consists in associating pegylated interferon and ribavirin, thrombocytopenia is also among the numerous side effects of this bitherapy, and the condition can potentially be induced or exacerbated during treatment [28]. It renders chronically infected patients either ineligible for such a treatment or eligible for an adapted course (reduced or discontinued doses), which can potentially decrease the probability of successful HCV treatment [27]. Some studies have shown a decreasing trend of platelet count with increasing fibrosis and cirrhosis among patients with chronic liver disease. Among these patients, the reduction in platelets is mainly caused by platelet destruction from splenic pooling, because of portal hypertension. Indirect evidence supports also the hypothesis of bone marrow suppression by HCV, partly mediated by a reduction of thrombopoetin (TPO) production due to liver cirrhosis and/or fibrosis [29-31]. However, in HCV infection, the virus itself or other mechanisms can also lead to low platelet counts [32-34]. Thrombocytopenia in HCV infection is complex and multiple mechanisms are involved. Besides the central decrease of megakaryocytosis due to TPO defects and possible direct viral suppression [35,36], increased peripheral immune-mediated destruction of platelets and hypersplenism may lead also to increased splenic platelet sequestration [29,37].

To explain the immune destruction of platelets, several hypotheses have been put forward: the HCV binding to platelet membrane (with consequent binding of anti-HCV antibody and phagocytosis of platelets), and an impairment of host immune system triggering the production of autoantibodies against platelet glycoproteins are the two most frequently postulated immune mechanisms explaining increased peripheral platelet destruction in HCV-infected cases [35,38-40]. Thrombocytopenia is now considered as a marker of HCV infection, which involves that all patients with low platelet count must be screened for the virus, especially in regions with high rates of infection [41,42].

In case of thrombocytopenia, the ultimate aim in HVC-positive patients is not to normalize the platelet counts [43], but to attain and maintain a safe hemostatic level that avoids hemorrhage on one hand and thrombosis on the other. Administration of Elthrombopag, a synthetic agonist of the TPO receptor that activates JAK2/STAT signaling pathways and induces increased proliferation and differentiation of human bone marrow progenitor cells into MKs and thus increased platelet production [44,45], has been proposed to meet this purpose [46,47].

### Platelets and dengue virus

Dengue is a tropical infectious disease caused by the single positive-stranded RNA virus of the *Flaviviridae* family, genus *Flavivirus*. It is now regarded as a possible threat in temperate climates where permissive mosquitoes are present [48,49]. It is always feared because of the possible severity of clinical symptoms (1% of cases) and because of the possibility that diseases evolves to a hemorrhagic form, with considerable drop in platelet count [50]. Even non-hemorrhagic dengue infections present with decreased numbers of circulating platelets [51]. In the recent past years, the role of platelets in regulating endothelial integrity and dengue-associated plasma leakage has gained increased interest [52-57]. Studies using platelet aggregometry showed reduced aggregation in dengue patients [58-60], but because the reliability of aggregometry is compromised in conditions with thrombocytopenia, these findings need to be interpreted with caution. There are, meanwhile, other platelet function alterations that are associated with plasma leakage [61]: dengue infection is generally associated with platelet activation with an increased expression of the activated fibrinogen receptor (αIIbβ3), the lysosomal marker CD63 and the alpha-granule marker CD62P (P-selectin). Indeed, Hottz et al. have reported a consistent increase of CD62P expression in isolated platelets from dengue patients [57]. The concentration of serotonin is increased in platelets of patients, perhaps as a consequence of platelet destruction or due to some upregulation of serotonin transporter (SERT), as it has been described to occur in association with activation of the αIIbβ3 integrin [62]. Upon maximal platelet activation by TRAP (*Thrombin* Receptor Activating Peptide), platelet function defects were observed with a significantly reduced maximal activated αIIbβ3 and CD63 expression and reduced platelet-monocyte and platelet-neutrophil complexes. Dengue infection stimulates platelets to produce pro-IL-1β, possibly *de novo*; mature IL-1β increases the production of PMPs, which themselves augment vascular permeability [56]. A molecular pattern for the binding of Dengue Virus on Platelets is DC-SIGN [63], the very same receptor that characterizes DV binding on the primary target i.e. dendritic cells [64]. This DV::platelet ligation enhances Phosphatidylserine expression by platelets—a signature of apoptosis—and a call for phagocytosis by macrophages [65].

Regarding platelets, dengue infection is not exempt of unexpected and somewhat paradoxical data: whereas Tsai et al. [66] and Onlamoon et al. [67] found an increase in platelet-leukocyte aggregates during dengue infection in humans and macaques, respectively, the circulating level of monocytes and polymorphonuclear cells was decreased. Further, at the febrile phase, interferon gamma-induced protein 10 (IP-10) is fairly detected in dengue patients with and without warning signs, and during defervescence as well; however, MIP-1β (Macrophage Inflammatory Protein-1 β) and MCP-1 (Monocyte chemotactic protein-1) are elevated only in patients with warning signs at this phase and G-CSF (Granulocyte colony-stimulating factor) is significant in patients without warning signs. There are further significant correlations between the levels of VEGF (vascular endothelial growth factor) , RANTES (regulated on activation, normal T cell expressed and secreted) , IL-7, IL-12, PDGF (Platelet-derived growth factor) and IL-5 with platelets; VEGF with lymphocytes and neutrophils; G-CSF and IP-10 with atypical lymphocytes and various other cytokines with the liver enzymes are reported [68]. In all, these data suggest complex interactions between virus, platelets and leukocytes. Because platelets and leukocytes are seminal to primary hemostasis and further to clotting, it may be envisioned, although experimental data is lacking, that those intertwined relationships are defective at a stage, allowing vessel leakage and hemorrhage.

The situation seems worsened because dengue virus infection is accompanied by a dampening of central megakaryogenesis. The down-regulation of haematopoiesis is probably a protective mechanism of the microenvironment that limits injury to the marrow stem/progenitor cell compartment during the subsequent process of elimination of infected cells [3].

Dengue fever is one of the rare infectious diseases that necessitates platelet component (PC) transfusion; PC transfusion is not systematic as infected individuals who do not bleed or present early signs of bleeding must not be transfused, even with a prophylactic perspective. PC transfusion is however indicated and must not be delayed in bleeding patients; this therapy has become very safe regarding the risk of transmission of other infectious agents at least in economically wealthy countries, and the immunological risk is kept minimal in occasional transfusion [69]. One must however be aware that dengue outbreaks pose threats to the PC inventory because the needs increase considerably while the number of healthy, non infected blood donor candidates is correlatively diminished, and because additional tests must be applied to detect viral markers (antigens and, recently, viral RNA). This also poses an economical burden to the public health systems.

### Platelets and arenaviruses

Several members of the *Arenaviridae* family are etiologic agents of viral hemorrhagic fevers (VHFs) in humans, a syndrome characterized by fever and bleeding complications that may ultimately lead to shock and death [70]. Different experimental models shed light on the role of platelets in VHF [50]. It was reported that mice infected with an acute strain (Armstrong) of lymphochoriomeningitis virus (LCMV), a prototype representative of *Arenaviridae* that was shown able to be episodically pathogen in the human species, developed a mild hemorrhagic anemia that became severe and eventually lethal in animals depleted of platelets or lacking integrin β3. In addition to the life-threatening hemorrhagic anemia, platelet-depleted mice failed to mount an efficient cytotoxic T lymphocyte (CTL) response and could not clear LCMV. Remarkably, lethal hemorrhage was less frequently associated with thrombocytopenia and instead was more closely associated with platelet dysfunction mediated by high type I interferon levels [71]. These observations confirm that platelets are necessary to protect vascular integrity and are critical mediators of viral clearance; they also outline the generally underscored and underappreciated relationships between primary hemostasis, viral infection and immunosuppression.

It is noteworthy that there are important differences between the LCMV mouse model for thrombocytopenia in which IFN-α/β mediates the decrease in platelet numbers, platelet dysfunction and bleeding, and the LCMV primate model, in which the IFN-α/β expression is not correlated to the presence of bleeding signs [71,72]. However, the role of type I IFN in mediating thrombocytopenia and platelet dysfunction was further supported by the infection of human CD34^+^ cells by Junin virus, an arenavirus that causes Argentinian Haemorrhagic Fever. In this disease, thrombopoiesis is selectively impaired by a decrease of pro-platelet formation, platelet release and P-selectin externalization [73]. This mechanism is supported by the recent observation that MKs express functional type I IFN receptors, suggesting that megakaryo/thrombopoiesis regulation by type I IFN is associated with a specific interaction with its receptor, and that these cells may play a role in the antiviral defense by being both type I IFN producers and responders [74].

Very recently, it was shown that mice profoundly depleted of platelets (<2.5%) and infected with the Armstrong LCMV strain developed systemic bleeding and hemorrhagic spots in several organs along with high viral titers, generalized splenic necrosis and increased mortality. Interestingly, the partial depletion (15%) of platelets was sufficient to prevent vascular damage but not viral replication, necrotic destruction of innate and adaptive immune splenocytes or CTL exhaustion [75]. The existence of different platelet requirements for controlling vascular integrity and immune response is an interesting novel concept, which suggests that these 2 events may be mediated by separated pathways at the platelet level. Moreover, these data give further support to the increasing evidence of the critical role of the physical interaction between platelets and immune cells to the host immune response during viral infection. However an important issue that requires further clarification is the molecular basis governing platelet interaction with immune cells in VHF.

The role of platelets in the immunosuppression occurring after infection by clone-13 of LMCV, a chronic, persistent strain, and treatment with TPO was also investigated [76]. Despite an increase in the number of platelets, no differences were seen in LCMV viral titers, indicating that other mechanisms mediate the deficient immune response seen in LCMV clone-13 infection. Interestingly the authors hypothesized that the selective inactivation of IFN-α/β signaling in MKs without altering their important antiviral activity in immune and stromal cells was found able to completely overcome the thrombocytopenia induced by infection with LCMV clone-13.

Among the different pathogenic mechanisms that account for the bleeding complications of VHF induced by arenaviruses, the presence of a platelet inhibitor has been reported in the serum of Lassa [77] and Junin virus infected-patients. This factor was not neutralized by plasma containing high titers of protective antibodies against Junin virus [78], suggesting that it is not a virus-derived molecule. Although this inhibitor has not yet been characterized, thrombomodulin could be a potential candidate. Indeed, thrombomodulin was shown to bind thrombin and block the ability of thrombin to activate platelets. Moreover, peripheral blood mononuclear cells (PBMC) exposed to a pathogenic Lassa virus increase expression of thrombomodulin mRNA. This pattern was observed also at the protein level in PBMC and dendritic cells for both soluble and membrane-bound thrombomodulin [50]. Thus, it is conceivable that the platelet aggregation inhibitor found in Lassa or Junin virus-infected patients is thrombomodulin, awaiting for laboratory confirmation.

Collectively, these data indicate that homeostatic abnormalities together with the antiviral immune response and high levels of viremia observed in arenavirus infection are partly regulated by platelet-virus interactions.

## Conclusion

In the light of the preceding considerations, the relationship between platelets and viruses appears to be very complex and heterogeneous. The commonest feature is probably the decrease of platelet count during viral infection, with sometimes threatening levels and bleeding. Two other common features are a profound disturbance of the coagulation process at large (primary hemostasis, clotting and fibrinolysis) and a sustained inflammation, some platelet factors intervening in both hemostasis and inflammation; a special link between these two events is very likely during viral infection but not yet fully understood. The functional properties of platelets are profoundly altered during viral infection and in particular their capacity to control the leukocyte containment within circulation or emigration to tissues; viral infections where platelet disturbances are noticeable seem to be accompanied by an altered vascular integrity, allowing leakage. The role of platelets as exerting virostatic effects would also need to be addressed; although it seems rather limited or at least over-helmed by the global inflammatory environment, the fact that platelets are induced to massive death as consequences of direct attacks of viruses or virus-infected cells must be taken into consideration. Finally, a better understanding of the interplay between platelets and viruses would certainly help correcting the platelet defects that are commonly observed and that may be severe, with no treatment identified so far; platelet component transfusions may be needed but, even though, with imprecise and non consensual recommendations for prescriptions and use. In aggregate, roles of viral platelets in viral infection are dominated by pathologic inflammation with evident but not exclusive consequences in hemostasis. Further research in platelet mediated-immunity during viral infection would be helpful, both from a conceptual point of view due to the variety of current pathophysiological situations and the likely severity of certain clinical pictures, especially when hemorrhagic symptoms are present.
